# Uneinheitliche Standards beim Tuberkulose‐Screening und der präventiven Tuberkulosetherapie vor Beginn einer systemischen Psoriasistherapie

**DOI:** 10.1111/ddg.15948_g

**Published:** 2026-06-04

**Authors:** Christoph Zeyen, Ruben Heuer, Brit Haecker, Tom Schaberg, Alexander Nast

**Affiliations:** ^1^ Division of Evidence‐Based Medicine (dEBM) Klinik für Dermatologie Venerologie und Allergologie Charité – Universitätsmedizin Berlin Mitglied der Freien Universität Berlin und der Humboldt‐Universität zu Berlin; ^2^ Deutsche Zentralkommission für Tuberkulosebekämpfung (DZK), Berlin

**Keywords:** IL‐17‐Antikörper, IL‐23‐Antikörper, Präventive Tuberkulosetherapie, Psoriasis, Tuberkulose, IL‐17 antibodies, IL‐23 antibodies, preventive tuberculosis therapy, psoriasis, tuberculosis

## Abstract

**Hintergrund und Ziele:**

Die präventive Therapie einer Tuberkulose (TB) vor der Einleitung von Methotrexat (MTX) oder IL‐17/IL‐23/IL‐12/23p40‐Inhibitoren bei latenter Tuberkuloseinfektion (LTBI) wird indirekt durch ein nachweislich erhöhtes TB‐Reaktivierungsrisiko unter TNF‐Inhibitoren gestützt. Direkte Nachweise für ein erhöhtes Risiko unter MTX oder IL‐17/IL‐23/IL‐12/23p40‐Inhibitoren fehlen jedoch. Zur besseren Einschätzung des TB‐Reaktivierungsrisikos sind Daten zu LTBI‐Patienten erforderlich, die diese Medikamente ohne präventive TB‐Therapie erhalten haben. Diese Studie wurde im Rahmen der Aktualisierung der europäischen und deutschen Psoriasis‐Leitlinien durchgeführt, um die aktuelle Praxis des LTBI‐Screenings und der präventiven TB‐Behandlung zu bewerten.

**Material und Methodik:**

Eine Online‐Umfrage wurde über deutsche, europäische und internationale dermatologische Fachgesellschaften und Netzwerke verteilt. Es gingen 326 vollständige Antworten ein.

**Ergebnisse:**

Ein LTBI‐Screening führen vor Einleitung von MTX 45% der Befragten durch, vor Therapie mit IL‐17/IL‐23/IL‐12/23p40‐Inhibitoren 95%. Eine präventive TB‐Therapie wird in 38% der LTBI‐Fälle vor MTX „immer“ oder „fast immer“ eingeleitet, in 31% „nie“ oder „fast nie“. Bei IL‐17/IL‐23/IL‐12/23p40‐Inhibitoren erfolgt die präventive Therapie in 66% der Fälle „immer“ oder „fast immer“, in 16% situationsabhängig, in 9% „nie“ oder „fast nie“.

**Schlussfolgerungen:**

Beim LTBI‐Screening sowie der präventiven TB‐Therapie vor MTX bestehen keine einheitlichen Standards. Für IL‐17/IL‐23/IL‐12/23p40‐Inhibitoren ist das Screening weit verbreitet, eine präventive TB‐Therapie wird dennoch bei einem relevanten Anteil der LTBI‐Patienten vor diesen Biologicals nicht verabreicht.

## EINLEITUNG

Ein erhöhtes Reaktivierungsrisiko einer latenten Tuberkuloseinfektion (LTBI) während der Behandlung mit Tumornekrosefaktor‐Inhibitoren (TNFi) ist belegt.^1^, ^2^ Eine erhebliche Anzahl aktiver Tuberkulosefälle wurde dokumentiert und berichtet.^3^, ^4^ Infolgedessen empfehlen wichtige Leitlinien ein LTBI‐Screening sowie eine präventive Tuberkulosetherapie (PT) vor Einleitung einer TNFi‐Therapie.^5^, ^6^, ^7^ Andererseits bestehen für konventionelle Psoriasistherapien wie Acitretin, Ciclosporin, Fumarsäureester sowie für das *Small Molecule* Apremilast keine derartigen Empfehlungen, da für diese Wirkstoffe kein erhöhtes Tuberkuloserisiko angenommen wird.^5^

Bei moderneren Biologika wie Interleukin (IL)‐17/IL‐23/IL‐12/23p40‐Inhibitoren oder dem *Small Molecule* Deucravacitinib orientieren sich viele Leitlinien an den Empfehlungen für TNFi und befürworten ebenfalls ein Screening und bei LTBI auch PT.^5^, ^6^ Diese Empfehlungen basieren jedoch hauptsächlich auf indirekter Evidenz, da Berichte über Tuberkulosereaktivierungen unter diesen Wirkstoffen selten sind^8^ und randomisierte Studien fehlen.^9^ Metaanalysen mit Beobachtungsdaten zeigen kein signifikant erhöhtes Reaktivierungsrisiko.^10^, ^11^ Ein Konsenspapier österreichischer Fachgesellschaften empfiehlt daher bei LTBI‐Patienten, die IL‐17‐Inhibitoren erhalten soll, auf eine PT zu verzichten, da das Risiko für Reaktivierung als gering eingeschätzt wird.^12^ Ein internationales Expertengremium des *Skin Inflammation and Psoriasis International Network* (SPIN) spricht sich für ein flexibleres Vorgehen aus. Es empfiehlt, bei LTBI auf PT zu verzichten, wenn IL‐17‐ oder IL‐23‐Inhibitoren oder Apremilast verordnet werden und das Risiko für Toxizität oder Wechselwirkungen der PT als hoch eingeschätzt wird.^11^ Auch bei geringem Risiko für Nebenwirkungen könnte laut SPIN auf PT verzichtet werden.^11^

Für Methotrexat (MTX) fehlen in verfügbaren Leitlinien häufig klare Empfehlungen zum Umgang mit LTBI, obwohl dieses Medikament häufig zur Behandlung der Psoriasis eingesetzt wird. Da MTX und IL‐17/IL‐23/IL‐12/23p40‐Inhibitoren auch in der Rheumatologie und Gastroenterologie verwendet werden, ist die Fragestellung fächerübergreifend von Relevanz.

Ein LTBI‐Screening erfolgt meist mit Interferon‐Gamma‐*Release‐Assays* (IGRA), oft kombiniert mit einer Thorax‐Röntgenaufnahme zum Ausschluss einer aktiven TB‐Erkrankung.^13^ Diese Diagnostik verursacht aber Kosten, ist nicht in jeder Einrichtung verfügbar und kann die Einleitung einer systemischen Therapie verzögern.^14^, ^15^ Wenn das Risiko einer Reaktivierung für bestimmte systemische Wirkstoffe als nicht erhöht eingestuft wird, könnten Kosten möglicherweise reduziert werden, was eine effizientere Ressourcenzuweisung ermöglicht. Außerdem könnte dies die Verordnung systemischer Psoriasistherapien für Ärzte ohne unmittelbaren Zugang zu Röntgendiagnostik beschleunigen, da sie ihre Patienten nicht zuerst in die Radiologie überweisen müssten.

Die Weltgesundheitsorganisation und die deutschen nationalen Tuberkulose‐Leitlinien empfehlen bei Bedarf eine PT nach einem der folgenden Schemata: drei Monate Isoniazid‐ und Rifampicin, vier Monate Rifampicin oder sechs bis neun Monate Isoniazid.^16^, ^17^ Der Beginn einer Biologika‐Behandlung gilt nach 4 bis 8 Wochen PT als sicher, obwohl diese Empfehlung nicht evidenzbasiert ist. Solche Verzögerungen können bei schwerer Psoriasis eine rechtzeitige Krankheitskontrolle behindern und sich negativ auf die Behandlungsergebnisse auswirken, insbesondere bei Personen mit hoher Krankheitslast oder begrenzten therapeutischen Alternativen. Nicht notwendige PT sollte vermieden werden, wenn die Risikobewertung dies zulässt, wobei die potenziellen Nebenwirkungen der Tuberkulostatika, wie Hepatotoxizität, Neurotoxizität und das Risiko von Arzneimittelwechselwirkungen, insbesondere bei Patienten, die mehrere Medikamente erhalten, zu berücksichtigen sind.^18^, ^19^

Um einen direkten Nachweis für ein erhöhtes Reaktivierungsrisiko – oder das Fehlen eines solchen – zu belegen, müssen die gemeldeten Fälle von TB‐Reaktivierung während der Einnahme von MTX und IL‐17/IL‐23/IL‐12/23p40‐Inhibitoren erfasst werden. Von besonderem Interesse sind Fälle, in denen Patienten diesen Medikamenten ausgesetzt waren, ohne sich einer PT zu unterziehen. Die gemeldeten Fälle müssen in Bezug auf die gesamte exponierte Patientenpopulation kontextualisiert werden. Die genaue Definition und das Verständnis dieser Patientenkohorte stellt jedoch eine große Herausforderung dar.

Wenn Dermatologen konsequent ein Screening durchführen und eine PT verabreichen, ist die Anzahl der beobachteten Reaktivierungsfälle möglicherweise sehr gering und das Reaktivierungsrisiko wird unterschätzt. Verabreichen Dermatologen dagegen häufig MTX und IL‐17/IL‐23/IL‐12/23p40‐Inhibitoren an LTBI‐Patienten ohne PT oder unterlassen sie das Screening von Hochrisikopopulationen mit LTBI, könnte die geringe Zahl der gemeldeten Fälle ein Zeichen für ein niedriges Reaktivierungsrisiko sein.

Diese Umfrage wurde im Rahmen der europäischen und deutschen Leitlinienentwicklung zur Aktualisierung der S3‐Leitlinie zur systemischen Psoriasisbehandlung durchgeführt. Das Hauptziel bestand darin, die derzeitigen Praktiken für das LTBI‐Screening und den Einsatz einer PT vor Beginn der systemischen Psoriasistherapien, insbesondere MTX und IL‐17/IL‐23/IL‐12/23p40‐Inhibitoren, zu bewerten, um den Anteil der Patienten mit Risiko für eine Tuberkulosereaktivierung zu schätzen. Bislang wurde in keiner Studie versucht, den Anteil der Patienten in diesem speziellen Zusammenhang zu ermitteln. Darüber hinaus wurde die derzeitige Praxis für andere Therapeutika parallel untersucht, darunter Acitretin, Ciclosporin, Fumarsäureester, Apremilast und Deucravacitinib.

## MATERIAL UND METHODIK

### Studiendesign

Diese explorative quantitative Querschnittsstudie wurde mittels einer anonymen Online‐Umfrage durchgeführt. Ein Umfragelink wurde über Mailinglisten deutscher dermatologischer Gesellschaften (Deutsche Dermatologische Gesellschaft (DDG), Berufsverband Deutscher Dermatologen (BVDD) und PSONET, Regionale Psoriasisnetze in Deutschland) sowie europäischer und internationaler Gesellschaften, darunter das *European Dermatology Forum (EDF)*, der *International Psoriasis Council* (IPC) und das *Skin Inflammation and Psoriasis International Network* (SPIN), an Dermatologen versandt.

Zur Gewährleistung methodischer Qualität und klinischer Relevanz wurde die Umfrage in enger Zusammenarbeit mit Experten aus der Dermatologie und Tuberkulosemedizin konzipiert. Die Umfrage umfasste Multiple‐Choice‐Fragen, Likert‐Skalen und offene Fragen, um Daten über die Praktiken und Einstellungen von Dermatologen zum LTBI‐Screening und zur PT zu sammeln.

Die Umfrage umfasste Fragen zur Praxis des LTBI‐Screenings vor Beginn systemischer antipsoriatischer Therapien, insbesondere MTX, IL‐17‐, IL‐23‐ und IL‐12/23p40‐Inhibitoren, sowie Acitretin, Ciclosporin, Fumarsäureester, Apremilast und Deucravacitinib. Die Behandler wurden auch gefragt, ob sie in Fällen von LTBI vor der Verabreichung jeder dieser antipsoriatischen Therapien eine PT einleiten.

Alle Dermatologen, die Zugang zu den Mailinglisten der Gesellschaften haben, konnten an der Umfrage teilnehmen. Das einzige Einschlusskriterium war die aktive Beteiligung an der Behandlung von Patienten mit Psoriasis. Demografische Informationen über die Befragten, wie zum Beispiel die Art der Einrichtung und die Psoriasis‐spezifische Expertise, wurden erhoben, um die Charakteristika der Stichprobe zu erfassen.

Während der gesamten Studie wurde sorgfältig darauf geachtet, ethische Standards einzuhalten und die Vertraulichkeit der erhobenen Daten zu gewährleisten.

### Datenerhebung

Die Datenerhebung erfolgte über einen Zeitraum von 2 Monaten, beginnend im November 2023, mit mindestens einer Erinnerungsmail etwa 4 Wochen nach dem ersten Versand. Die Antworten wurden anonymisiert erfasst.

LimeSurvey Community Edition Version 6.2.1+230807 wurde sowohl für den Entwurf als auch für die Durchführung der Umfrage verwendet.

### Datenanalyse

Die Datenanalyse beinhaltete die Zusammenfassung der Umfrageergebnisse, die Berechnung von Anteilen sowie die Identifikation von Trends und Mustern im dermatologischen Behandlungsverhalten. Deskriptive Statistiken wurden verwendet, um die Häufigkeit und Verteilung der LTBI‐Screening‐Praktiken sowie den Anteil der Dermatologen zu bestimmen, die PT vor der Einleitung systemischer antipsoriatischer Therapien verabreichen. Die statistischen Analysen wurden mit R Version 4.3.1 durchgeführt (http://www.r‐project.org).

### Ethik

Die Umfrage wurde von der lokalen Ethikkommission der Charité Berlin genehmigt (Antragsnummer EA4/208/23).

## ERGEBNISSE

Insgesamt wurden circa 8785 Dermatologen per E‐Mail‐Verteiler über verschiedene Mailinglisten kontaktiert. Davon wurden 7165 (81,56%) über deutsche Kanäle erreicht, während 1620 (18,44%) über internationale Kanäle kontaktiert wurden.

Insgesamt beantworteten 326 Dermatologen die Umfrage vollständig, was einer Rücklaufquote von 3,71% entspricht. Es wurden 147 (45,09%) Antworten über deutsche Verteiler, 179 (54,91%) über internationale Verteiler erzielt. Die einzelnen Rücklaufquoten und alle folgenden Ergebnisse pro Verteiler sind im Online‐Appendix 1–4 aufgelistet.

Alle Befragten waren klinisch tätige Dermatologen. Davon waren 309 (94,79%) Fachärzte, 17 (5,21%) in der Facharztweiterbildung. Die Ärzte wurden gebeten, ihre Kompetenz in der Psoriasis‐Behandlung auf einer Skala von 0 (keine Kompetenz) bis 10 (maximale Kompetenz) selbst einzuschätzen. Insgesamt 92,02% der Befragten stuften ihre Fachkenntnisse mit 7 oder höher ein, also im oberen Drittel der vorgegebenen Skala.

Von den befragten Dermatologen gaben 146 (44,79%) an, in einer Praxis zu arbeiten, während 174 (53,37%) berichteten, in einem Klinikumfeld tätig zu sein. Weitere sechs Befragte (1,84%) gaben an, in anderen Bereichen als diesen beiden zu arbeiten.

### Tuberkulose‐Screening‐Maßnahmen

Von allen Befragten gaben 50,61% an, sowohl IGRA als auch Röntgenaufnahmen des Thorax zum Screening durchzuführen, während 35,28% ausschließlich auf IGRA zurückgreifen. Zusätzlich gaben 14,11% an, „andere“ Screening‐Maßnahmen zu verwenden, die nicht näher spezifiziert wurden. Es ist anzunehmen, dass in den meisten dieser Fälle ein Tuberkulin‐Hauttest durchgeführt wird.

### MTX, TNFi und IL‐17/IL‐23/IL‐12/23p40‐Inhibitoren – LTBI‐Screening

MTX, TNFi sowie IL‐17/IL‐23/IL‐12/23p40‐Inhibitoren wurden von den europäischen und deutschen Psoriasis‐Leitliniengruppen als zentrale Fokus‐Therapien identifiziert, da unter Experten von einer kontroversen Bewertung ausgegangen wurde. Die Ergebnisse zu diesen Wirkstoffen werden daher getrennt von denen zu konventionellen Therapien und *Small Molecules* berichtet.

**ABBILDUNG 1:**
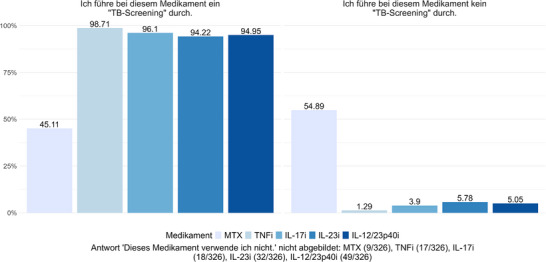
Screening auf latente Tuberkuloseinfektion (LTBI) vor Beginn einer Psoriasistherapie – Antworten auf die Frage: „Vor der Einleitung welches der folgenden Medikamente führen Sie ein TB‐Screening durch?“

Die Teilnehmenden wurden gefragt: „Vor Einleitung welches der folgenden Medikamente führen Sie ein TB‐Screening durch?“ Vor Beginn einer immunmodulatorischen Psoriasistherapie mit MTX führen 45,11% der Dermatologen ein LTBI‐Screening durch, während 54,89% in diesen Fällen kein Screening veranlassen. Vor Einleitung einer Therapie mit TNF‐Inhibitoren gaben 98,71% der Befragten an, ein LTBI‐Screening durchzuführen; 1,29% verzichten darauf. Vor Therapiebeginn mit IL‐17‐, IL‐23‐ oder IL‐12/23p40‐Inhibitoren wird in der Mehrzahl der Fälle ein LTBI‐Screening vorgenommen (96,1% für IL‐17‐Inhibitoren, 94,22% für IL‐23‐Inhibitoren und 94,95% für IL‐12/23p40‐Inhibitoren). In einem relevanten Anteil der Fälle erfolgt jedoch kein Screening (Abbildung 1).

### MTX, TNFi und IL‐17/IL‐23/IL‐12/23p40‐Inhibitoren – Präventive Therapie bei LTBI

Die Expertinnen und Experten wurden gefragt:

„Sie haben einen Patienten vor sich mit einer latenten Tuberkuloseinfektion (positiver Quantiferon‐Test) und einer unauffälligen Röntgen‐Thoraxaufnahme/aktive Tuberkulose wurde ausgeschlossen. Es besteht eine Indikation zur Einleitung einer systemischen Psoriasis‐Therapie. Es liegen keine weiteren Risikofaktoren vor, die eine TB‐Reaktivierung fördern würden. Bei welcher der folgenden Therapien führen Sie eine präventive Tuberkulosetherapie (zum Beispiel mit Rifampicin [4 Monate] oder Isoniazid + Rifampicin [3 Monate] oder Isoniazid [9 Monate]) durch?“

Für MTX wird in 37,54% der LTBI‐Fälle eine präventive Therapie („immer“ oder „fast immer“) durchgeführt. In 31,23% der Fälle erfolgt die präventive Therapie „nie“ oder „fast nie“ vor Beginn der MTX‐Behandlung. 21,14% der Befragten entscheiden situationsabhängig gemeinsam mit den Patienten über die Durchführung einer PT.

Bei TNFi erfolgt in 79,55% der LTBI‐Fälle eine vorbeugende Therapie („immer“ oder „fast immer“). 5,11% gaben an, „nie“ oder „fast nie“ eine PT durchzuführen. 5,75% entscheiden fallweise nach gemeinsamer Entscheidungsfindung mit den Patienten.

**ABBILDUNG 2:**
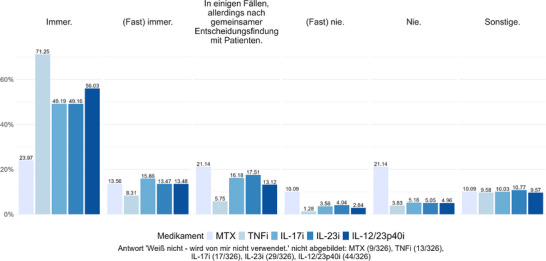
Präventive Tuberkulosetherapie vor Beginn einer Psoriasistherapie – Antworten auf die Frage: „Bei welcher der folgenden Therapien führen Sie eine präventive Tuberkulosetherapie (zum Beispiel mit Rifampicin [4 Monate] oder Isoniazid + Rifampicin [3 Monate] oder Isoniazid [9 Monate]) durch?“

Die Verteilung der Antworten zur Durchführung einer PT vor Einleitung einer Therapie mit IL‐17‐, IL‐23‐ oder IL‐12/23p40‐Inhibitoren ist in Abbildung 2 dargestellt. Insgesamt wird in etwa zwei Dritteln der Fälle („immer“ oder „fast immer“) eine PT durchgeführt. Bemerkenswerterweise erfolgt in 8,74% der Fälle mit IL‐17‐Inhibitoren, in 9,09% der Fälle mit IL‐23‐Inhibitoren und in 7,8% der Fälle mit IL‐12/23p40‐Inhibitoren „nie“ oder „fast nie“ eine präventive Therapie.

### Konventionelle Therapien und *small molecules*

Während MTX, TNFi sowie IL‐17‐, IL‐23‐ und IL‐12/23p40‐Inhibitoren im Fokus der Leitliniengruppen standen, wurde im Rahmen dieser Untersuchung auch der derzeitige Behandlungsstandard für konventionelle Therapien und neu zugelassene *Small Molecules* untersucht. Für diese Wirkstoffe wurde zunächst ein geringeres Maß an Kontroverse unter den Experten erwartet.

Für konventionelle Psoriasistherapien wie Acitretin, Ciclosporin und Fumarsäureester sowie das *Small Molecule* Apremilast empfehlen weder Leitlinien noch Fachinformationen präventive Tuberkulosetherapie.^5^, ^20^ Im Gegensatz dazu wird für das kürzlich zugelassene *Small Molecule* Deucravacitinib (2023) ein LTBI‐Screening sowie eine präventive Therapie empfohlen – unter anderem aufgrund der noch begrenzten *Real‐World*‐Datenlage.

Die Umfrageergebnisse zu diesen Substanzen werden im vorliegenden Manuskript separat dargestellt. Eine Übersicht ist in den Tabellen 1 und 2 zu finden.

**TABELLE 1:** Screening vor Einleitung konventioneller Psoriasistherapien und *small molecules*.

LTBI‐Screening vor Einleitung von	LTBI‐Screening n (%)	Kein LTBI‐Screening n (%)	Dieses Medikament verwende ich nicht n (%)
Acitretin	13 (3,99)	279 (85,58)	34 (10,43)
Ciclosporin	95 (29,14)	183 (56,13)	48 (14,72)
Fumarsäureester	17 (5,21)	191 (58,59)	118 (36,2)
Apremilast (PDE‐4‐Inhibitor)	79 (24,23)	162 (49,69)	85 (26,07)
Deucravacitinib (TYK2‐Inhibitor)	154 (47,24)	15 (4,6)	157 (48,16)

**TABELLE 2:** Präventive Tuberkulosetherapie vor Einleitung konventioneller Psoriasistherapien und *small molecules*.

Präventive TB‐Therapie vor Einleitung von …	Immer n (%)	(Fast) immer n (%)	In einigen Fällen, allerdings nach gemeinsamer Entscheidungsfindung mit Patienten n (%)	(Fast) nie n (%)	Nie n (%)	Sonstige n (%)
Acitretin	18 (6,1)	1 (0,34)	24 (8,14)	35 (11,86)	195 (66,1)	22 (7,46)
Ciclosporin	58 (21,09)	30 (10,91)	50 (18,18)	30 (10,91)	78 (28,36)	29 (10,55)
Fumarsäureester	15 (7,46)	6 (2,99)	17 (8,46)	21 (10,45)	122 (60,7)	20 (9,95)
Apremilast (PDE‐4‐Inhibitor)	38 (15,38)	13 (5,26)	33 (13,36)	41 (16,6)	96 (38,87)	26 (10,53)
Deucravacitinib (TYK2‐Inhibitor)	85 (49,71)	23 (13,45)	18 (10,53)	6 (3,51)	14 (8,19)	25 (14,62)

## DISKUSSION

Die Ergebnisse der Umfrage verdeutlichen eine ausgeprägte Heterogenität beim LTBI‐Screening sowie der Durchführung präventiver TB‐Therapie vor Einleitung systemischer Psoriasistherapien. Bemerkenswert ist, dass nur etwa die Hälfte der Behandelnden sowohl einen IGRA als auch eine Röntgenaufnahme des Thorax zur Diagnostik durchführt, während die andere Hälfte ausschließlich einen IGRA nutzt. Diese Diskrepanz könnte auf logistische Herausforderungen bei der Überweisung von Patienten zur Röntgenuntersuchung zurückzuführen sein, die zu erheblichen Verzögerungen beim Behandlungsbeginn führen können. Es ist wichtig zu beachten, dass IGRA‐Ergebnisse falsch‐negativ ausfallen können – insbesondere bei Patienten, die bereits eine immunsuppressive Therapie erhalten.^21^

Daher sollte ein LTBI‐Screening – sofern indiziert – vor Beginn vor allem einer TNF‐Inhibitor‐Therapie erfolgen, um das Risiko falsch‐negativer Ergebnisse zu minimieren. Es wird berichtet, dass der ELISpot‐Test bei Patienten mit aktiver Tuberkulose und nicht eindeutigen Testergebnissen eine leicht höhere Sensitivität als der Quantiferon‐Test aufweisen könnte.^22^ Aktuelle Tuberkulose‐Leitlinien identifizieren spezifische Risikogruppen mit erhöhter Wahrscheinlichkeit für eine TB und empfehlen in diesen Fällen sogar explizit die Kombination aus IGRA und Thorax‐Röntgen zur Diagnostik.^7^, ^17^

Die Ergebnisse zur Frage, bei welchen antipsoriatischen Therapien ein LTBI‐Screening vorausgeht, sind insbesondere im Fall von MTX interessant: Hier wird bei fast der Hälfte der Patienten ein Screening durchgeführt, während die andere Hälfte ohne vorherige Diagnostik behandelt wird. Die hohe Screeningrate vor Einleitung von TNFi war zu erwarten, während die etwas niedrigeren Raten bei IL‐17‐, IL‐23‐ und IL‐12/23p40‐Inhibitoren möglicherweise das als geringer eingeschätzte Reaktivierungsrisiko unter diesen Wirkstoffen widerspiegeln.

Die Ergebnisse zur präventiven TB‐Therapie korrelieren eng mit den Screeningraten: Etwa ein Drittel der LTBI‐positiven Patienten erhält eine PT vor Einleitung von MTX. Das ist bemerkenswert, da die Fachinformation zu MTX eine solche Maßnahme nicht empfiehlt. Die PT‐Raten vor Einleitung von TNF‐Inhibitoren sind deutlich höher, was zu dem weit verbreiteten Konsens über das erhebliche Reaktivierungsrisiko unter diesen Wirkstoffen passt. Ähnlich zum Screening sind die PT‐Raten bei IL‐17‐, IL‐23‐ und IL‐12/23p40‐Inhibitoren niedriger, was auf eine geringere Besorgnis hinsichtlich Reaktivierungen schließen lässt.

In der klinischen Praxis werden viele Biologika‐naive Patienten zunächst mit einem Adalimumab‐Biosimilar behandelt, bedingt durch wirtschaftliche Anreize oder Vorgaben zur Erstattungsfähigkeit. In solchen Fällen wird ein LTBI‐Screening bereits zu Beginn verpflichtend, unabhängig davon, ob später ein Therapiewechsel erfolgt. Dies kann in Ländern mit entsprechenden Einschränkungen einen pragmatischen, sequenziellen Ansatz unterstützen: Durchführung eines LTBI‐Screenings vor Einleitung einer systemischen Therapie, Behandlung mit Adalimumab bei negativem Screeningbefund oder nach Beginn einer PT. Ist eine PT kontraindiziert, kann eine Therapie mit IL‐17‐ oder IL‐23‐Inhibitoren ohne PT erwogen werden. Diese Strategie könnte in künftigen Leitlinienaktualisierungen weiter ausgearbeitet werden. Im Gegensatz dazu gelten in Ländern, in denen IL‐17‐, IL‐23‐ oder IL‐12/23p40‐Inhibitoren routinemäßig als systemische Erstlinientherapien eingesetzt werden, potenziell andere Rahmenbedingungen.

Bei Patienten mit bekannter LTBI, die bereits eine präventive Therapie oder Behandlung einer aktiven Tuberkulose erhalten haben, ist das Reaktivierungsrisiko reduziert – eine wiederholte PT kann gegebenenfalls entfallen. Wenn möglich, können zur weiteren Minimierung des TB‐Risikos dennoch andere Wirkstoffe als TNFi in Betracht gezogen werden.

Ein weiteres bemerkenswertes Ergebnis ist die hohe Variabilität im Umgang mit konventionellen Therapien, die ursprünglich als unstrittig galten, da sowohl Leitlinien als auch Fachinformationen klare Empfehlungen geben. Trotz gegenteiliger Leitlinienempfehlungen und fehlender Hinweise auf ein erhöhtes Reaktivierungsrisiko führen knapp 30% der Behandler ein LTBI‐Screening vor Beginn einer Ciclosporin‐Therapie durch, rund 24% vor Start einer Behandlung mit Apremilast. Zudem verordnen über 30% der Dermatologen potenziell nebenwirkungsreiche Tuberkulostatika vor Ciclosporin und über 20% vor Apremilast.

Zurückgehend auf den ursprünglichen Ansatz, den Anteil von LTBI‐Patienten zu schätzen, die MTX, TNFi oder IL‐17/IL‐23/IL‐12/23p40‐Inhibitoren ohne vorbeugende Therapie erhalten, lassen sich mehrere Schlussfolgerungen ziehen:

Weniger als 50% der Behandler führen vor Beginn einer MTX‐Therapie überhaupt ein Screening durch. Zudem beginnen weniger als 40% in Fällen mit positivem LTBI‐Screening „immer“ oder „fast immer“ eine PT. Folglich erhält ein erheblicher Anteil der LTBI‐Patienten vermutlich MTX ohne vorbeugende Therapie.

Die Raten von Behandlern, die IL‐17‐, IL‐23‐ oder IL‐12/23p40‐Inhibitoren ohne vorheriges Screening und/oder ohne PT einsetzen, sind zwar nicht hoch, jedoch verzichten etwa 3%–5% auf das Screening und 6%–8% führen keine PT durch. Vor diesem Hintergrund ist die Zahl potenziell exponierter Patienten relevant und es wäre zu erwarten, dass einzelne Reaktivierungsfälle beobachtet werden.

Unter Berücksichtigung der Epidemiologie, Prävalenz und Inzidenz von Tuberkulose und LTBI sowie der tausenden Patientenjahre unter Exposition gegenüber bestimmten Biologika, wäre im Falle eines tatsächlich erhöhten Reaktivierungsrisikos mit einer entsprechenden Zahl von Reaktivierungen zu rechnen. Es wird geschätzt, dass 20%–25% der Weltbevölkerung von LTBI betroffen sind,^23^, ^24^ mit einer Prävalenz von 11%–14% in Europa.^23^, ^24^ Ohne immunsuppressive Therapie wird das lebenslange Risiko einer Reaktivierung bei Personen mit unbehandelter LTBI auf etwa 5%–10% geschätzt.^25^, ^26^, ^27^ Wird eine immunsuppressive Therapie verabreicht, treten Reaktivierungen am wahrscheinlichsten innerhalb der ersten 6 Monate nach Therapiebeginn auf,^28^ was sie relativ frühzeitig nachweisbar macht. Angesichts dieser bekannten Raten würde ein signifikanter Anstieg an Reaktivierungsfällen aller Wahrscheinlichkeit nach in klinischen Daten oder Fallberichten zu beobachten sein.

Die Tatsache, dass nahezu alle teilnehmenden Ärzte Fachärzte für Dermatologie waren und über 90% ihre Psoriasis‐bezogene Expertise mit ≥ 7/10 einschätzten, untermauert die Aussagekraft der Ergebnisse zusätzlich. Die Entscheidung, auf ein Screening oder eine PT zu verzichten, scheint daher bewusst getroffen worden zu sein, geleitet von klinischer Erfahrung und nicht von einem Mangel an Wissen.

### Limitationen

Da die Datenerhebung anonym erfolgte, kann nicht ausgeschlossen werden, dass einzelne Personen die Umfrage mehrfach beantwortet haben.

Die Umfrage erfasste keine Informationen zu patientenspezifischen Merkmalen wie Demografie, Krankheitsaktivität, Komorbidität oder weiteren Risikofaktoren, die das Vorgehen beim LTBI‐Screening und bei der Durchführung einer PT beeinflussen könnten.

Die Rücklaufquote von etwa 4% könnte zu einem Non‐Response‐Bias führen und die Generalisierbarkeit der Ergebnisse einschränken. Es ist denkbar, dass vor allem Dermatologen mit besonderem Interesse an Psoriasis teilgenommen haben, während Kollegen mit breiterem, weniger spezialisierten Tätigkeitsfeld unterrepräsentiert sind. Dennoch verleiht der hohe Grad an Psoriasis‐spezifischer Expertise der Teilnehmer den Ergebnissen Glaubwürdigkeit. Auch wenn die Auswahl über spezifische Mailinglisten potenziell einzelne Gruppen ausschloss, spricht die ausgewogene Verteilung der Befragten zwischen niedergelassenem Bereich (etwa die Hälfte) und Klinik für eine gute Abdeckung des gesamten dermatologischen Versorgungsspektrums.

Eine weitere Einschränkung betrifft die geografische Verteilung der Teilnehmer, die primär in Deutschland lag, ergänzt durch Rückmeldungen aus weiteren europäischen und internationalen Ländern. Obwohl 179 Antworten von nichtdeutschen Teilnehmern vorlagen, reichte diese Zahl nicht aus, um aussagekräftige länderspezifische Analysen durchzuführen. Daher konnten potenzielle regionale Unterschiede in der Tuberkuloseinzidenz, den nationalen Gesundheitssystemen oder der klinischen Praxis nicht vollständig berücksichtigt werden. Diese Aspekte schränken die Übertragbarkeit der Ergebnisse auf Länder mit abweichender epidemiologischer oder gesundheitspolitischer Ausgangslage ein.

Zudem wurde keine Erhebung zu wiederholtem oder routinemäßigem LTBI‐Screening vorgenommen, was insbesondere bei Patienten unter langfristiger immunsuppressiver Therapie, vor allem in Ländern mit hoher Tuberkuloseinzidenz, relevant sein könnte.

Die Zahl der täglich behandelten Patienten pro Teilnehmer kann stark variieren.

Insgesamt bestätigen subtile, aber durchgängig reproduzierte Unterschiede zwischen TNFi und den anderen Biologika das gute Verständnis der Thematik und die Qualität der erhobenen Daten.

### Schlussfolgerungen

Unter Dermatologen besteht eine erhebliche Heterogenität im Vorgehen beim LTBI‐Screening und bei der präventiven Tuberkulosetherapie vor Einleitung einer systemischen Psoriasisbehandlung.

Auffällig ist die Variabilität im diagnostischen Vorgehen: Während einige Behandler ausschließlich den IGRA verwenden, kombinieren andere den IGRA mit einer Thorax‐Röntgenaufnahme. LTBI‐Screenings werden zwar routinemäßig vor Beginn einer Therapie mit IL‐17‐, IL‐23‐ und IL‐12/23p40‐Inhibitoren durchgeführt, vor MTX jedoch deutlich uneinheitlicher. Auch PT wird vor Beginn einer Behandlung mit IL‐17‐, IL‐23‐ und IL‐12/23p40‐Inhibitoren häufig eingeleitet. Ein Teil der Dermatologen verfolgt bei diesen Wirkstoffen jedoch ein individualisiertes Vorgehen bei Screening und PT, sodass ein gewisser Anteil von Patienten diesen Psoriasistherapien ohne begleitende präventive TB‐Therapie ausgesetzt ist.

Die in dieser Studie dargestellten Ergebnisse unterstreichen die Notwendigkeit einer Weiterentwicklung der dermatologischen Leitlinien auf deutscher wie europäischer Ebene, um langfristig ein einheitlicheres Vorgehen zu fördern. Das SPIN‐Positionspapier liefert bereits einen konsensbasierten Rahmen für den Verzicht auf PT bei LTBI‐Patienten unter IL‐17‐ oder IL‐23‐Inhibitoren. Künftige Forschung sollte sich auf LTBI‐Patienten konzentrieren, die Wirkstoffen mit bislang unklarem Reaktivierungsrisiko ausgesetzt sind – idealerweise im Rahmen eines multinationalen Registers.

Multidisziplinäre Leitlinien zum LTBI‐Screening und zur PT sollten regelmäßig auf Basis neuer Evidenz aktualisiert werden. Eine ausgewogene Abwägung zwischen Nutzen und Risiken der PT ist ebenso wünschenswert wie eine wirtschaftlich tragfähige und praxisnahe Umsetzung im jeweiligen Gesundheitssystem.

## DANKSAGUNG

Wir danken den folgenden Organisationen für ihre Unterstützung: *Skin Inflammation & Psoriasis International Network* (SPIN), *International Psoriasis Council* (IPC), *European Dermatology Forum* (EDF), Prof. M. Augustin für die regionalen Psoriasisnetze in Deutschland (PsoNet), Deutsche Dermatologische Gesellschaft (DDG) und Berufsverband der Deutschen Dermatologen (BVDD).

Wir danken Martin Dittmann für seine redaktionelle Unterstützung.

Open access Veröffentlichung ermöglicht und organisiert durch Projekt DEAL.

## INTERESSENKONFLIKT

Keiner.

## Supporting information



Supplementary information
