# Supplementary material for: Uneinheitliche Standards beim Tuberkulose‐Screening und der präventiven Tuberkulosetherapie vor Beginn einer systemischen Psoriasistherapie
Source: J Dtsch Dermatol Ges. 2026 Jun 4;24(6):737–45. [Article in German] doi: 10.1111/ddg.15948_g (PMC13238406; doi:10.1111/ddg.15948_g)
Supplement: Supplementary file 1 — Supplementary information [file DDG-24-737-s001.docx]

# Anhang

**Anhang 1:** Antworten nach Verteiler

|  | **Alle Verteiler n (%)** | **BVDD n (%)** | **DDG* n (%)** | **PsoNet n (%)** | **EDF n (%)** | **IPC n (%)** | **SPIN n (%)** |
| --- | --- | --- | --- | --- | --- | --- | --- |
| Anzahl Antworten | **8785 (100)** | 3421 (100) | 3635 (100) | 109 (100) | 253 (100) | 1217 (100) | 150 (100) |
| Anzahl vollständige Antworten | **326 (3,71)** | 122 (3,57) | 0 (0) | 25 (22,94) | 23 (9,09) | 94 (7,72) | 62 (41,33) |
| *Über den DDG-Mailverteiler wurden keine Rückmeldungen erhalten. Auf die Umfrage, die hinter einer Login-Schranke lag, wurde nicht zugegriffen. | | | | | | | |

**Anhang 2:** LTBI-Screening-Methoden nach Verteilern

| **Screeningmethoden** | **Alle Verteiler n (%)** | **BVDD n (%)** | **DDG* n (%)** | **PsoNet n (%)** | **EDF n (%)** | **IPC n (%)** | **SPIN n (%)** |
| --- | --- | --- | --- | --- | --- | --- | --- |
| IGRA + Röntgen-Thorax | **165 (50,61)** | 69 (56,56) | 0 (0) | 17 (68) | 9 (39,13) | 41 (43,62) | 29 (46,77) |
| Interferon-Gamma Release Assay (IGRA), z.B. Quantiferon-Test | **115 (35,28)** | 52 (42,62) | 0 (0) | 7 (28) | 11 (47,83) | 26 (27,66) | 19 (30,65) |
| Andere | **46 (14,11)** | 1 (0,82) | 0 (0) | 1 (4) | 3 (13,04) | 27 (28,72) | 14 (22,58) |
| **Total** | **326 (100)** | **122 (100)** | **0 (100)** | **25 (100)** | **23 (100)** | **94 (100)** | **62 (100)** |
| *Über den DDG-Mailverteiler wurden keine Rückmeldungen erhalten. Auf die Umfrage, die hinter einer Login-Schranke lag, wurde nicht zugegriffen. | | | | | | | |

**Anhang 3:** LTBI-Screening vor Einleitung einer systemischen Psoriasistherapie nach Verteiler

| **„Vor der Einleitung welches der folgenden Medikamente führen Sie ein „TB-Screening“ durch?“** | | **Alle Verteiler** | | **BVDD** | | **DDG*** | | **PsoNet** | | **EDF** | | **IPC** | | **SPIN** | |
| --- | --- | --- | --- | --- | --- | --- | --- | --- | --- | --- | --- | --- | --- | --- | --- |
|  |  | **Ja n(%)** | **Nein n(%)** | **Ja n(%)** | **Nein n(%)** | **Ja n(%)** | **Nein n(%)** | **Ja n(%)** | **Nein n(%)** | **Ja n(%)** | **Nein n(%)** | **Ja n(%)** | **Nein n(%)** | **Ja n(%)** | **Nein n(%)** |
| Konventionelle Therapien | Acitretin | **13 (4,45)** | **279 (95,55)** | 5  (4,76) | 100 (95,24) | 0 (0) | 0 (0) | 0  (0) | 23  (100) | 1 (4,55) | 21 (95,45) | 3 (3,57) | 81 (96,43) | 4  (6,9) | 54 (93,1) |
|  | Ciclosporin | **95 (34,17)** | **183 (65,83)** | 20 (22,73) | 68 (77,27) | 0 (0) | 0 (0) | 6  (27,27) | 16  (72,73) | 5 (22,73) | 17 (77,27) | 32 (36,78) | 55 (63,22) | 32 (54,24) | 27 (45,76) |
|  | Fumarsäureester | **17 (8,17)** | **191 (91,83)** | 9  (7,5) | 111 (92,5) | 0 (0) | 0 (0) | 0  (0) | 25 (100) | 0  (0) | 15 (100) | 2 (9,09) | 20 (90,91) | 6 (23,08) | 20 (76,92) |
|  | Methotrexat | **143 (45,11)** | **174 (54,89)** | 63 (52,94) | 56 (47,06) | 0 (0) | 0 (0) | 15  (60) | 10  (40) | 4 (18,18) | 18 (81,82) | 34 (37,36) | 57 (62,64) | 27  (45) | 33 (55) |
| Biologicals | TNF-Inhibitoren | **305 (98,71)** | **4 (1,29)** | 118 (99,16) | 1 (0,84) | 0 (0) | 0 (0) | 25 (100) | 0  (0) | 22 (100) | 0  (0) | 82 (98,80) | 1  (1,2) | 58 (96,67) | 2 (3,33) |
|  | IL-17-Inhibitoren | **296 (96,1)** | **12 (3,9)** | 119 (98,35) | 2 (1,65) | 0 (0) | 0 (0) | 24  (96) | 1  (4) | 20 (90,91) | 2  (9,09) | 77 (95,06) | 4 (4,94) | 56 (94,92) | 3 (5,08) |
|  | IL-23-Inhibitoren | **277 (94,22)** | **17 (5,78)** | 118 (97,52) | 3 (2,48) | 0 (0) | 0 (0) | 24  (96) | 1  (4) | 18 (85,71) | 3 (14,29) | 68 (93,15) | 5 (6,85) | 49 (90,74) | 5 (9,26) |
|  | Ustekinumab  (IL-12/23p40) | **263 (94,95)** | **14 (5,05)** | 106 (98,15) | 2 (1,85) | 0 (0) | 0 (0) | 25 (100) | 0  (0) | 18 (85,71) | 3 (14,29) | 65 (94,20) | 4 (5,80) | 49 (90,74) | 5 (9,26) |
| Small Molecules | Apremilast  (Phosphodiesterase-4-Inhibitor) | **79 (32,78)** | **162 (67,22)** | 39 (37,14) | 66 (62,86) | 0 (0) | 0 (0) | 4  (18,18) | 18  (81,82) | 1 (6,25) | 15 (93,75) | 20 (36,36) | 35 (63,64) | 15 (34,88) | 28 (65,12) |
|  | Deucravacitinib  (TYK2-Inhibitor) | **154 (91,12)** | **15 (8,88)** | 77 (93,90) | 5  (6,10) | 0 (0) | 0 (0) | 16  (100) | 0  (0) | 10 (83,33) | 2  (16,67) | 30 (93,75) | 2 (6,25) | 21 (77,78) | 6 (22,22) |
| “Dieses Medikament verwende ich nicht” nicht enthalten. | | | | | | | | | | | | | | | |
| *Über den DDG-Mailverteiler wurden keine Rückmeldungen erhalten. Auf die Umfrage, die hinter einer Login-Schranke lag, wurde nicht zugegriffen. | | | | | | | | | | | | | | | |

**Anhang 4:** Präventive TB-Therapie im Fall von LTBI vor Einleitung einer systemischen Psoriasistherapie nach Verteiler

|  | **Präventive TB-Therapie vor Einleitung einer systemischen Psoriasistherapie – Antworten auf die Frage „Bei welcher der folgenden Therapien führen Sie eine präventive TB-Therapie (z.B. mit Rifampicin (4 Monate) oder Isoniazid+Rifampicin (3 Monate) oder Isoniazid (9 Monate)) durch?“** | | **Alle Verteiler  n (%)** | **BVDD n (%)** | **DDG^*^ n (%)** | **PsoNet n (%)** | **EDF n (%)** | **IPC n (%)** | **SPIN n (%)** |
| --- | --- | --- | --- | --- | --- | --- | --- | --- | --- |
| Konventionelle Therapien | Acitretin | Nie. | **195 (66,1)** | 67 (66,34) | 0 (0) | 18 (75) | 15 (71,43) | 57 (64,04) | 38 (63,33) |
|  |  | (Fast) nie. | **35 (11,86)** | 8 (7,92) | 0 (0) | 4 (16,67) | 2 (9,52) | 13 (14,61) | 8 (13,33) |
|  |  | In einigen Fällen, allerdings nach gemeinsamer Entscheidungsfindung mit Patienten. | **24 (8,14)** | 7 (6,93) | 0 (0) | 1 (4,17) | 3 (14,29) | 8 (8,99) | 5 (8,33) |
|  |  | (Fast) immer. | **1 (0,34)** | 0 (0) | 0 (0) | 0 (0) | 0 (0) | 1 (1,12) | 0 (0) |
|  |  | Immer. | **18 (6,1)** | 3 (2,97) | 0 (0) | 1 (4,17) | 1 (4,76) | 8 (8,99) | 5 (8,33) |
|  |  | Andere. | **22 (7,46)** | 16 (15,84) | 0 (0) | 0 (0) | 0 (0) | 2 (2,25) | 4 (6,67) |
|  | Ciclosporin | Nie. | **78 (28,36)** | 35 (40,7) | 0 (0) | 6 (30) | 9 (45) | 17 (19,1) | 11 (18,33) |
|  |  | (Fast) nie. | **30 (10,91)** | 8 (9,3) | 0 (0) | 3 (15) | 1 (5) | 13 (14,61) | 5 (8,33) |
|  |  | In einigen Fällen, allerdings nach gemeinsamer Entscheidungsfindung mit Patienten. | **50 (18,18)** | 9 (10,47) | 0 (0) | 4 (20) | 4 (20) | 20 (22,47) | 13 (21,67) |
|  |  | (Fast) immer. | **30 (10,91)** | 7 (8,14) | 0 (0) | 1 (5) | 2 (10) | 12 (13,48) | 8 (13,33) |
|  |  | Immer. | **58 (21,09)** | 10 (11,63) | 0 (0) | 3 (15) | 4 (20) | 22 (24,72) | 19 (31,67) |
|  |  | Andere. | **29 (10,55)** | 17 (19,77) | 0 (0) | 3 (15) | 0 (0) | 5 (5,62) | 4 (6,67) |
|  | Fumarsäureester | Nie. | **122 (60,7)** | 74 (62,18) | 0 (0) | 17 (68) | 9 (75) | 9 (39,13) | 13 (59,09) |
|  |  | (Fast) nie. | **21 (10,45)** | 11 (9,24) | 0 (0) | 4 (16) | 0 (0) | 6 (26,09) | 0 (0) |
|  |  | In einigen Fällen, allerdings nach gemeinsamer Entscheidungsfindung mit Patienten. | **17 (8,46)** | 8 (6,72) | 0 (0) | 2 (8) | 2 (16,67) | 2 (8,7) | 3 (13,64) |
|  |  | (Fast) immer. | **6 (2,99)** | 4 (3,36) | 0 (0) | 1 (4) | 0 (0) | 0 (0) | 1 (4,55) |
|  |  | Immer. | **15 (7,46)** | 5 (4,2) | 0 (0) | 1 (4) | 1 (8,33) | 5 (21,74) | 3 (13,64) |
|  |  | Andere. | **20 (9,95)** | 17 (14,29) | 0 (0) | 0 (0) | 0 (0) | 1 (4,35) | 2 (9,09) |
|  | MTX | Nie. | **67 (21,14)** | 27 (23,08) | 0 (0) | 4 (16) | 9 (42,86) | 16 (17,39) | 11 (17,74) |
|  |  | (Fast) nie. | **32 (10,09)** | 12 (10,26) | 0 (0) | 1 (4) | 1 (4,76) | 11 (11,96) | 7 (11,29) |
|  |  | In einigen Fällen, allerdings nach gemeinsamer Entscheidungsfindung mit Patienten. | **67 (21,14)** | 16 (13,68) | 0 (0) | 7 (28) | 6 (28,57) | 21 (22,83) | 17 (27,42) |
|  |  | (Fast) immer. | **43 (13,56)** | 11 (9,4) | 0 (0) | 4 (16) | 3 (14,29) | 18 (19,57) | 7 (11,29) |
|  |  | Immer. | **76 (23,97)** | 28 (23,93) | 0 (0) | 7 (28) | 2 (9,52) | 23 (25) | 16 (25,81) |
|  |  | Andere. | **32 (10,09)** | 23 (19,66) | 0 (0) | 2 (8) | 0 (0) | 3 (3,26) | 4 (6,45) |
| Biologicals | TNF-Inhibitoren | Nie. | **12 (3,83)** | 8 (6,72) | 0 (0) | 0 (0) | 0 (0) | 4 (4,65) | 0 (0) |
|  |  | (Fast) nie. | **4 (1,28)** | 3 (2,52) | 0 (0) | 0 (0) | 0 (0) | 0 (0) | 1 (1,61) |
|  |  | In einigen Fällen, allerdings nach gemeinsamer Entscheidungsfindung mit Patienten. | **18 (5,75)** | 9 (7,56) | 0 (0) | 1 (4) | 3 (14,29) | 3 (3,49) | 2 (3,23) |
|  |  | (Fast) immer. | **26 (8,31)** | 10 (8,4) | 0 (0) | 3 (12) | 2 (9,52) | 7 (8,14) | 4 (6,45) |
|  |  | Immer. | **223 (71,25)** | 65 (54,62) | 0 (0) | 19 (76) | 16 (76,19) | 70 (81,4) | 53 (85,48) |
|  |  | Andere. | **30 (9,58)** | 24 (20,17) | 0 (0) | 2 (8) | 0 (0) | 2 (2,33) | 2 (3,23) |
|  | IL-17-Inhibitoren | Nie. | **16 (5,18)** | 9 (7,5) | 0 (0) | 1 (4) | 1 (4,76) | 4 (4,88) | 1 (1,64) |
|  |  | (Fast) nie. | **11 (3,56)** | 3 (2,5) | 0 (0) | 2 (8) | 0 (0) | 4 (4,88) | 2 (3,28) |
|  |  | In einigen Fällen, allerdings nach gemeinsamer Entscheidungsfindung mit Patienten. | **50 (16,18)** | 21 (17,5) | 0 (0) | 3 (12) | 6 (28,57) | 9 (10,98) | 11 (18,03) |
|  |  | (Fast) immer. | **49 (15,86)** | 12 (10) | 0 (0) | 5 (20) | 4 (19,05) | 12 (14,63) | 16 (26,23) |
|  |  | Immer. | **152 (49,19)** | 52 (43,33) | 0 (0) | 13 (52) | 10 (47,62) | 48 (58,54) | 29 (47,54) |
|  |  | Andere. | **31 (10,03)** | 23 (19,17) | 0 (0) | 1 (4) | 0 (0) | 5 (6,1) | 2 (3,28) |
|  | IL-23-Inhibitoren | Nie. | **15 (5,05)** | 10 (8,26) | 0 (0) | 1 (4) | 1 (4,76) | 2 (2,7) | 1 (1,79) |
|  |  | (Fast) nie. | **12 (4,04)** | 3 (2,48) | 0 (0) | 2 (8) | 0 (0) | 6 (8,11) | 1 (1,79) |
|  |  | In einigen Fällen, allerdings nach gemeinsamer Entscheidungsfindung mit Patienten. | **52 (17,51)** | 21 (17,36) | 0 (0) | 2 (8) | 7 (33,33) | 8 (10,81) | 14 (25) |
|  |  | (Fast) immer. | **40 (13,47)** | 9 (7,44) | 0 (0) | 7 (28) | 5 (23,81) | 8 (10,81) | 11 (19,64) |
|  |  | Immer. | **146 (49,16)** | 55 (45,45) | 0 (0) | 12 (48) | 8 (38,1) | 44 (59,46) | 27 (48,21) |
|  |  | Andere. | **32 (10,77)** | 23 (19,01) | 0 (0) | 1 (4) | 0 (0) | 6 (8,11) | 2 (3,57) |
|  | Ustekinumab (IL-12/23p40) | Nie. | **14 (4,96)** | 9 (8,18) | 0 (0) | 0 (0) | 1 (5) | 4 (5,63) | 0 (0) |
|  |  | (Fast) nie. | **8 (2,84)** | 5 (4,55) | 0 (0) | 0 (0) | 0 (0) | 2 (2,82) | 1 (1,79) |
|  |  | In einigen Fällen, allerdings nach gemeinsamer Entscheidungsfindung mit Patienten. | **37 (13,12)** | 16 (14,55) | 0 (0) | 1 (4) | 6 (30) | 7 (9,86) | 7 (12,5) |
|  |  | (Fast) immer. | **38 (13,48)** | 8 (7,27) | 0 (0) | 9 (36) | 5 (25) | 8 (11,27) | 8 (14,29) |
|  |  | Immer. | **158 (56,03)** | 52 (47,27) | 0 (0) | 14 (56) | 8 (40) | 46 (64,79) | 38 (67,86) |
|  |  | Andere. | **27 (9,57)** | 20 (18,18) | 0 (0) | 1 (4) | 0 (0) | 4 (5,63) | 2 (3,57) |
| Small Molecules | Apremilast | Nie. | **96 (38,87)** | 44 (41,12) | 0 (0) | 9 (42,86) | 8 (47,06) | 21 (37,5) | 14 (30,43) |
|  |  | (Fast) nie. | **41 (16,6)** | 15 (14,02) | 0 (0) | 4 (19,05) | 2 (11,76) | 8 (14,29) | 12 (26,09) |
|  |  | In einigen Fällen, allerdings nach gemeinsamer Entscheidungsfindung mit Patienten. | **33 (13,36)** | 9 (8,41) | 0 (0) | 4 (19,05) | 5 (29,41) | 8 (14,29) | 7 (15,22) |
|  |  | (Fast) immer. | **13 (5,26)** | 5 (4,67) | 0 (0) | 1 (4,76) | 1 (5,88) | 3 (5,36) | 3 (6,52) |
|  |  | Immer. | **38 (15,38)** | 14 (13,08) | 0 (0) | 1 (4,76) | 1 (5,88) | 14 (25) | 8 (17,39) |
|  |  | Andere. | **26 (10,53)** | 20 (18,69) | 0 (0) | 2 (9,52) | 0 (0) | 2 (3,57) | 2 (4,35) |
|  | Deucravacitinib | Nie. | **14 (8,19)** | 11 (13,25) | 0 (0) | 0 (0) | 1 (11,11) | 2 (5,56) | 0 (0) |
|  |  | (Fast) nie. | **6 (3,51)** | 3 (3,61) | 0 (0) | 1 (6,67) | 0 (0) | 0 (0) | 2 (7,14) |
|  |  | In einigen Fällen, allerdings nach gemeinsamer Entscheidungsfindung mit Patienten. | **18 (10,53)** | 9 (10,84) | 0 (0) | 1 (6,67) | 2 (22,22) | 3 (8,33) | 3 (10,71) |
|  |  | (Fast) immer. | **23 (13,45)** | 6 (7,23) | 0 (0) | 2 (13,33) | 3 (33,33) | 8 (22,22) | 4 (14,29) |
|  |  | Immer. | **85 (49,71)** | 35 (42,17) | 0 (0) | 9 (60) | 3 (33,33) | 20 (55,56) | 18 (64,29) |
|  |  | Andere. | **25 (14,62)** | 19 (22,89) | 0 (0) | 2 (13,33) | 0 (0) | 3 (8,33) | 1 (3,57) |
| “Dieses Medikament verwende ich nicht” nicht enthalten. | | | | | | | | | |
| *Über den DDG-Mailverteiler wurden keine Rückmeldungen erhalten. Auf die Umfrage, die hinter einer Login-Schranke lag, wurde nicht zugegriffen. | | | | | | | | | |
